# Perspectives and controversies regarding the use of natural products for the treatment of lung cancer

**DOI:** 10.1002/cam4.3660

**Published:** 2021-03-02

**Authors:** Tingting Wen, Lei Song, Shucheng Hua

**Affiliations:** ^1^ Department of Respiratory Medicine Key Laboratory of Organ Regeneration & Transplantation of the Ministry of Education The First Hospital of Jilin University Changchun Jilin P.R. China

**Keywords:** apoptosis, chemotherapy, lung cancer, nanoparticles, natural products

## Abstract

Lung cancer is the leading cause of cancer‐related mortality both in men and women and accounts for 18.4% of all cancer‐related deaths. Although advanced therapy methods have been developed, the prognosis of lung cancer patients remains extremely poor. Over the past few decades, clinicians and researchers have found that chemical compounds extracted from natural products may be useful for treating lung cancer. Drug formulations derived from natural compounds, such as paclitaxel, doxorubicin, and camptothecin, have been successfully used as chemotherapeutics for lung cancer. In recent years, hundreds of new natural compounds that can be used to treat lung cancer have been found through basic and sub‐clinical research. However, there has not been a corresponding increase in the number of drugs that have been used in a clinical setting. The probable reasons may include low solubility, limited absorption, unfavorable metabolism, and severe side effects. In this review, we present a summary of the natural compounds that have been proven to be effective for the treatment of lung cancer, as well as an understanding of the mechanisms underlying their pharmacological effects. We have also highlighted current controversies and have attempted to provide solutions for the clinical translation of these compounds.

## Graphical abstract

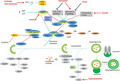

## 1 INTRODUCTION

Malignant tumors are a major public problem that seriously threatens human health. Lung cancer has the highest fatality rate, which has caused it to be the leading cause of cancer‐related death.^1^ It has been estimated that 2.1 million new lung cancer cases and 1.8 million deaths were reported worldwide in 2018, equivalent to 18.4% of all cancer‐related deaths.^2^ Lung cancer can be divided into two main types: non‐small cell lung cancer (NSCLC) and small cell lung cancer (SCLC). NSCLC accounts for 85% of all lung cancer cases^3^ and has been further categorized into three subgroups based on its histology: squamous cell carcinoma, adenocarcinoma, and large cell carcinoma.^4^

Modes of treatment available for lung cancer include radiotherapy, chemotherapy, surgery, immunotherapy, and targeted therapy. Surgery and radiotherapy are usually used to treat early‐stage lung cancer patients, but these treatment modes are associated with a high risk of cancer recurrence.^5^ At present, the most used chemotherapeutic drugs are carboplatin and paclitaxel. However, severe adverse reactions and drug resistance are still the main obstacles for successful treatment when using these drugs.^6^ The discovery of epidermal growth factor receptor (EGFR) mutations has promoted the development of targeted molecular therapies, such as tyrosine kinase inhibitors (TKIs). It has widely reshaped the cancer treatment paradigm of NSCLC. However, in most cases, these types of therapies have been found to eventually develop resistance to first and second generation TKI.^7^ Immune checkpoint inhibitors (ICIs) can significantly impact clinical outcomes in several types of solid tumors, including NSCLC. Still, several clinical and biological barriers need to be overcome, and associated predictive markers need to be identified.^8^ Despite the development of alternative interventions, the results of their use have been unsatisfactory, with the prognosis being poor and the combined five‐year survival rate for all stages reaching only about 16%.^2^ Therefore, it is necessary to identify more effective methods of treatment that present fewer disadvantages.

Natural substances have been extensively used for the prevention and treatment of various diseases since ancient times. Paclitaxel, which is currently used for cancer treatment, is derived from plants and is used as an antimicrotubular drug that enhances the role of the tubulin dimer and stabilizes microtubules, while inhibiting its disassembly from promoting microtubule assembly and inhibiting cell replication.^9^ The Food and Drug Administration (FDA) has approved its use in combination with cisplatin for patients with AIDS‐related Kaposi's sarcoma, ovarian and breast cancer, and patients who cannot undergo surgery with/without radiotherapy for NSCLC. It has also been clinically used to treat recurrent or refractory SCLC.^10^ This has triggered an upsurge in the study of plant‐derived compounds. Along with exploring a variety of organisms and applying new technologies, potential bioactive compounds extracted from natural products have received an increased amount of attention regarding their use in cancer treatment. Some of these compounds, such as ginsenoside Rg3 and topotecan (a derivative of camptothecin), have already been used in a clinical setting, while others, such as luteic acid and silybin, are still undergoing clinical trials. These compounds have been found to exert many anticancer activities on lung cancer cells, and these anticancer effects involve autophagy, apoptosis, and a variety of signal pathways.

Hundreds of natural products have been proven to inhibit the development of tumors. The complications that arise from the use of these natural products have limited their large‐scale clinical application, while the advancement of nanotechnology and other emerging technologies have increased their potential effectiveness. This study summarizes the mechanisms, prospects, and challenges for the clinical application of natural products and their derivatives to treat lung cancer.

## 2 ANTINEOPLASTIC DRUGS CLASSIFIED BY SOURCE

### 2.1 Plants

#### 2.1.1 Ginsenosides

Ginsenosides are frequently used in East Asia and North America. Ginsenosides, the main components extracted from ginseng, exert various bioactivities, such as anti‐tumor, immunomodulatory, antioxidant produce, and anti‐inflammation effects.^11^ Ginsenosides mainly exert their anticancer effects on lung, breast, liver, and colorectal cancers. Ginsenoside Rg3, Rh2, and compound K are the main bioactive compounds in ginsenosides that produce anticancer effects.^12^ Among them, ginsenoside Rg3 has been approved as an anticancer drug by the China Food and Drug Administration (CFDA) in 2000 and was listed as a designated drug for the treatment of NSCLC in the Clinical Practice Guide of the National Comprehensive Cancer Network (Chinese version) in 2006 and 2007.^13^ Protopanaxadiol (PPT) and Propanaxadiol (PPD), two metabolites of ginsenosides, have also shown activity against a variety of cancer cells while PPT is more effective in inhibiting the viability and invasiveness of lung cancer cells, especially lung squamous cells.^11^

#### 2.1.2 Camptothecin (CPT)

Camptothecin (CPT) was the first natural compound derived from *Camptotheca acuminata*. It is a quinoline alkaloid that was first synthesized by Wall and Wani in 1966.^14^ Topotecan, a derivative of camptothecin, has been used as a first‐line and second‐line chemotherapeutic drug for SCLC. In the European Union and the United States, it is considered as the only drug suitable for use as a second‐line chemotherapeutic drug for recurrent SCLC. Irinotecan in combination with cisplatin has been used for the treatment of SCLC.^15^ Recently published *in vitro* and *in vivo* studies on CPT and its derivatives, such as irinotecan (CPT‐11, 4), Belotecan (CKD‐602, 5), and 10‐hydroxycamptothecin (HCPT), have reported that they exert a wide range of anti‐tumor activities on multiple types of tumors, including ovarian cancer, NSCLC, and refractory colorectal cancer.^16^, ^17^, ^18^ Belo is a relatively new camptothecin derivative approved in Korea for the treatment of NSCLC and ovarian cancers. Compared with older camptothecin preparations, belotecan has been reported to show a similar effective level and a decreased toxic level.^19^ Several other camptothecin analogs, including 7‐(4‐methylpiperazinomethylene)‐10, 9‐aminocamptothecin, exatecan mesylate, 11‐ethylenedioxy‐20(S)‐camptothecin, 9‐nitrocamptothecin, and karenitecin, are also at various stages of clinical development.^20^ However, the side effects of their use include diarrhea, fatigue, myelosuppression, stomatitis, nausea, vomiting, abdominal pain, hair loss, and peripheral neuropathy.^21^

#### 2.1.3 Curcumin (CUR)

Turmeric is a spice originally found in India used in curries and as a natural colorant. It contains three bioactive polyphenols: curcumin (CUR), demethoxycurcumin (DMC), and bisdemethoxycurcumin (BMC).^22^ In a paper published in 1949 in the Journal Nature, Schraufstatter and his colleagues reported that curcumin is a bioactive compound with antibacterial properties that are active against a variety of bacterial strains.^23^ Curcumin is also known to exert antioxidant and anticancer effects and is important for the treatment or prevention of various diseases, such as cardiovascular diseases and diabetes.^24^ Its anticancer activity was confirmed in 1980 by Kuttan and his colleagues using *in vitro* and *in vivo* models.^23^ Zhang et al. first proved that curcumin exerts anticancer effects using human lung adenocarcinoma cells with multidrug resistance to A549/DDP.^25^

#### 2.1.4 β‐elemene (β‐ELE)

β‐elemene is a natural sesquiterpene extracted from turmeric, a traditional Chinese herbal medicine, and is a non‐cytotoxic II antineoplastic drug.^26^ β‐elemene has been approved to treat many types of cancers, including brain, breast, prostate, ovarian, and lung cancer, with no severe side effects being reported. It can inhibit the migration, invasion, proliferation and enhance the radiosensitivity of lung cancer cells.^27^

#### 2.1.5 Gambogic (GA)

GA is a natural product extracted from Han's Geng Huang resin. Since ancient times, it has been used as a detoxification, anti‐inflammatory and anti‐parasitic drug in China and Southeast Asia.^28^ The use of GA, which has the molecular formula C38H44O9 (628.34 g/mol),^29^ is advantageous since it produces low levels of toxicity, resistance to many cell lines and multiple mechanisms, and is a potential anti‐tumor compound. The National Medical Products Administration has approved GA for use in treating advanced lung, liver, stomach, breast and colon cancers after the successful conclusion of clinical trials.^30^ The CFDA has also approved it for clinical trials for the therapy of various other types of cancers.^31^

#### 2.1.6 Tanshinone

*Salvia miltiorrhiza* has high medicinal value in traditional Chinese medicine (TCM) and can be used to treat a variety of cardio‐cerebrovascular diseases, including angina pectoris, myocardial infarction, hyperlipidemia, hypertension, and acute ischemic stroke.^32^ Recent studies have shown that *Salvia miltiorrhiza* is an effective inhibitor of platelet agglutination. Additionally, clinical trials have shown that *Salvia miltiorrhiza* is also effective for treating and preventing Alzheimer's disease.^33^ Nakao et al. isolated tanshinone from *Salvia miltiorrhiza* for the first time since the 1930s, and since then, more than 90 derived chemical constituents have been identified. These compounds can be divided into compounds with over 40 lipophilic components and compounds with over 50 hydrophilic compounds.^34^ Among them, the proportion of tanshinone IIA (TSA, Tan IIA) is the highest. Accumulated evidence has shown that tanshinone IIA exerts a broad‐spectrum of anti‐tumor properties against various malignant human tumors.^32^

#### 2.1.7 Licorice chalcone (LIC)

Licorice is a traditional Chinese medicinal drug widely used in clinical settings. Its main function involves the regulation of temperature, moisturization of the lungs, alleviation of toxicity, and coordination of the properties of drugs.^35^ It is used to treat gastritis, ulceration, coughs, bronchitis, and inflammation.^36^ In addition to triterpenes, about 300 polyphenols, including phenolic acids, flavonoids, flavanes, chalcones, and isoflavones, with a concentration of 1%–5% each, have been isolated from licorice root and licorice extract.^37^ Among them, licorice chalcone A (LICA), Licochalcone B (LCB), and Licochalcone D (LCD) have been proven to provide a variety of health benefits, including anticancer effects.^38^, ^39^, ^40^, ^41^

#### 2.1.8 Triptolide (TPL)

The root extract of *Tripterygium wilfordii* has been used to treat various diseases throughout history. TPL was first isolated from the Chinese herbal medicine *Tripterygium wilfordii* in 1972^42^ and is a diterpene lactone compound that contains three epoxy groups and is the main active component of *Tripterygium wilfordii*. Therefore, the discovery of TPL has paved the way for further anti‐tumor studies on triptolide.^43^ TPL can inhibit cancer cell growth and exhibit preclinical anti‐tumor activity on many types of cancers, including neuroblastoma, lung cancer, breast cancer, acute myeloid leukemia (AML), osteosarcoma, ovarian cancer, prostate cancer, and multiple gastrointestinal cancers (e.g., cancers of the stomach, liver, colon, and pancreas).^42^

#### 2.1.9 Emodin (ED)

Emodin is a natural active anthraquinone compound extracted from the rhubarb rhizome.^44^ Pharmacological studies have shown that emodin exerts a wide variety of activity, including anti‐inflammation and anti‐tumor effects, and prevents the development of many health issues, such as lung injury, pancreatitis, intestinal mucosal injury, and ulcerative colitis.^45^ Previous studies have shown that emodin exerts its antiproliferative effects on various cancer cells, including lung cancer, pancreatic cancer, breast cancer, colorectal cancer, leukemia, and hepatocellular carcinoma.^46^ Emodin can inhibit the growth and apoptosis of A549 cells through its action on the external apoptosis pathway and the induction of cell cycle arrest.^47^

##### Berberine (BBR)

*Coptis chinensis* is a traditional Chinese medicinal drug of high value that is commonly used in China. Berberine is the primary bioactive component of *Coptis chinensis*, accounting for 5.20%–7.69% of all compounds.^48^ It exerts various pharmacological effects, including antioxidant, anti‐microbial, liver protection, anti‐inflammatory, anti‐tumor, neuroprotection, blood lipid, and hypoglycemic effects.^49^ Recent studies have indicated that BBR exerts its anticancer effects on several high‐risk cancers, including lung, prostate, colorectal, breast, and gastric cancer.^50^ Additionally, other *in vitro* studies on tumor cell lines have shown that BBR inhibits cancer cell proliferation and migration and induces the apoptosis of a variety of cancer cell lines.^51^

#### 2.1.10 Epigallocatechin gallate (EGCG)

Green tea is a popular non‐alcoholic drink in Asian countries, and its long‐term consumption has been found to provide many health benefits. A 10‐year prospective cohort study conducted in Japan found that drinking 10 cups of green tea (120 ml/cup) a day delayed cancer onset.^52^ Catechin is the main active ingredient in green tea. The main catechins are (‐)‐epicatechin gallate (ECG), (‐)‐epicatechin (EC), (‐)‐epigallocatechin (EGC), catechin, and (‐)‐epigallocatechin‐3‐gallate (EGCG).^53^ EGCG is the main catechin found in green tea and accounts for 50‐‐80% of the total quantity of catechins. EGCG exerts antioxidant activity and affects a variety of human diseases, including Parkinson's disease, Alzheimer's disease, diabetes, stroke and obesity.^54^ Animal and cell line studies have shown that EGCG plays a crucial role in promoting apoptosis and reducing cancer growth, and is potential chemoprophylaxis and therapeutic compound for skin cancer, prostate cancer, lung cancer, colon cancer, breast cancer, and other cancers.^55^

#### 2.1.11 Resveratrol (RSV)

Resveratrol (RSV) is a naturally occurring polyphenol that is commonly found in red wine, peanuts, and grapes. It has been found to exert numerous biological properties, including antioxidant, antifungal, neuroprotective, anti‐inflammatory, antiviral, and anticancer properties.^56^ The most well‐known property of RSV is its antioxidation property, which can convert free radicals, such as reactive oxygen species (ROS), into inactive compounds.^57^ *In vitro* and *in vivo* studies conducted on resveratrol have found that hat it can be used for the therapy of a wide variety of human cancers, including lung, skin, breast, blood, cervical, and bone cancers, as well as gastrointestinal tumors.^58^

#### 2.1.12 Artemisinin

In 1972, a team led by Tu Youyou first isolated artemisinin through a plant screening research program called Project 523, which aimed to identify suitable antimalarial treatment drugs. In 2015, the discovery of artemisinin earned China its first Nobel Prize winner.^59^ It has been reported that artemisinin‐based drugs, such as dihydroartemisinin, artemether, artemisinin, and artesunate, are highly effective active antimalarial drugs that also exert effective anticancer activities against hematological tumors and somatic tumors, and selective cytotoxicity toward malignant cells.^60^ Therefore, they have become research hotspots. Studies have found that artemisinin exerts a variety of pharmacological effects against inflammation, viral infection, and cell and tumor proliferation, and has been shown to have a relatively safe toxicity profile, indicating the ability of artemisinin to decrease inflammation, invasion and metastasis, proliferation, and induce apoptosis.^61^

#### 2.1.13 Silybin

Silybin (C25H22O10), the main bioactive ingredient of silymarin, is a compound isolated from the fruits and seeds of silymarin. It has a long history of human use and does not exert apparent toxicity in animals and humans even after a long period of usage. It is commonly used as a nutritional and healthy food in Europe, Asia, and the United States.^62^ The antioxidant and hepatoprotective effects of silybin have led to its use to treat acute and chronic liver diseases caused by drugs, toxins, hepatitis, and alcohol, as well as gallbladder diseases.^63^ Preclinical studies have shown that silybin can strongly inhibit the invasion and migration of cancer cells.^64^ Its anti‐tumor activity has been demonstrated in animal models of cancers of the liver, lung, skin, prostate, and colon.^65^ Phase Ⅰ/Ⅱ clinical trials of prostate cancer are currently being conducted, and phase Ⅰ studies have reported it to be non‐toxic.^66^

#### 2.1.14 Cinnamon

Cinnamon is a spice and traditional herbal medicine used for hundreds of years that has been shown to exert antioxidant and free radical scavenging properties.^67^ Available evidence has shown that cinnamon exerts anti‐tumor, anti‐inflammatory, anti‐microbial, cholesterol‐lowering, antioxidant, and immunomodulatory effects. It can also act as an insulin mimic that can enhance insulin activity or stimulate cell glucose metabolism ^68^ and affect apoptosis.^69^ The cinnamon extract contains several active ingredients, such as essential oils (cinnamaldehyde), tannin, caryophyllene, cinnamyl acetate, linalool, and eugenol.^70^, ^71^ Whole cinnamon and its active components have been reported to exert significant anti‐tumor activity in different types of cancer.^72^

### 2.2 Animals

#### 2.2.1 Astaxanthin (ATX)

Astaxanthin (ATX) is a ketone carotene widely found in shrimp, salmon, crab, red yeast, and other marine animals.^73^ It exerts strong antioxidant activity, anti‐tumor effects, anti‐inflammatory effects, and hepatoprotective effects.^74^ In 1987, ATX was approved by the USFDA as a feed additive for aquaculture and was subsequently approved as a dietary supplement in 1999.^75^ In addition, numerous evidence has shown that ATX exerts anticancer effects on many types of cancers, including lung cancer, colon cancer, lung cancer, and breast cancer.^73^, ^76^

#### 2.2.2 Melittin

Bee venom is a complex mixture of bioactive substances that can be injected into specific parts of the body during bee venom therapy. In recent years, bee venom has been used to treat many diseases, such as cancer, chronic pain, arthritis and atopic dermatitis.^77^ Melittin is the main component of the venom and is an amphiphilic peptide with 26 amino acid residues.^78^ It exerts radiation protective, anti‐mutagenicity, and anti‐inflammation and analgesic activities.^79^ In addition, previous studies have already proven that melittin can induce cell cycle arrest, growth inhibition, and apoptosis of a variety of cancer cells, and has become an ideal anticancer candidate due to its broad‐spectrum of lytic properties.^80^

#### 2.2.3 Snake venom

Although snakebite poisoning is a public health problem that can endanger human lives, snake venom is considered a potential source of bioactive compounds. Snake wine or snake venom wine is used in conventional Chinese medicine.^81^ Snake venom is primarily a complex mixture of proteins with enzyme activity, with the proteins and peptides accounting for about 90% of the dry weight of the venom.^82^ These enzymes include metalloproteinase (MP), L‐amino acid oxidase (LAAOs), integrin, C lectin, and phospholipase A2 (PLA2 s).^83^ Their mechanisms of action include direct toxicity (PLA2 s), induction of cell apoptosis (PLA2 s, LAAOs, and MP), free radical production (LAAOs), and anti‐angiogenesis activity (double integrin and lectin). These compounds exert higher levels of cytotoxicity and cell inhibitory activity on tumor cells than that exerted on normal cells, which indicates the potential clinical application of snake venom.^84^

#### 2.2.4 Scorpion venom

Scorpions are among the most primitive arthropods of the animal kingdom that have existed on the earth for more than 400 million years and are globally distributed. Since ancient times, scorpion venom has been used in traditional medicinal applications of different countries, especially in China, India, Cuba, Africa, and Spain.^85^ Scorpion venom is a complicated mixture of proteins (peptides and enzymes) and non‐proteins (nucleotides, lipids, free amino acids, inorganic salts, and water).^86^ The most important components of scorpion venom are disulfide bridging peptides (DBPs/DBs) and non‐disulfide bridging peptides (NDBPs/NDBs), which exert a variety of pharmacological activities and have been used for the therapy of many diseases, including cancer.^87^ Their specific interactions with ion channels may be the main mechanism by which the anticancer activity of scorpion venom is exerted.^88^

### 2.3 Microorganisms

#### 2.3.1 Doxorubicin (Dox)

Doxorubicin, an antibiotic derived from *Streptomyces peucetius* bacteria, is an anthracycline chemotherapeutic drug. Since the 1960 s, it has been widely used as a chemotherapeutic agent.^89^ It was approved for use in the US in 1974 and is still an important drug used in many cancer chemotherapy programs.^21^ It is commonly used for the treatment of lung cancer, gastric cancer, soft tissue sarcoma, thyroid cancer, multiple myeloma, breast cancer, bladder cancer, ovarian cancer, and Hodgkin's lymphoma.^90^ However, its use produces serious side effects, with the most prominent being cardiotoxicity that can lead to left ventricular insufficiency, dilated cardiomyopathy, and heart failure, and the severity of heart disease is related to the accumulated dose of Dox administered during anticancer treatment.^91^

#### 2.3.2 Staurosporine (STS)

Staurosporine was first isolated from *Streptomyces* and has since then been isolated from other actinomycetes after the screening of microbial alkaloids by the Kitasato Research Institute of Japan in 1977.^92^ It is an effective inhibitor of PKC and many other kinases (including tyrosine protein kinases), which prevent the transfer of phosphate from DNA to the activated tyrosine site, which directly inhibits the activity of topoisomerase II.^93^ It can also induce apoptosis of many types of cells, including various cancer cells.^94^

#### 2.3.3 Epothilone

Epothilone is a natural cytotoxic compound extracted from myxobacteria and *Cysticercus cellulosus*.^95^ Similar to taxane, epomycin can induce the formation of microtubule bundles and inhibit microtubule dynamics, resulting in the inhibition of cell proliferation and mitotic arrest.^96^ Its activity is 10–1000 times higher than that of paclitaxel, and most importantly, unlike paclitaxel, ebolomycin can easily be used in combination with other drugs for the treatment of cancer.^97^ Atpresent, phase I‐III clinical trials are being conducted on five types of ebomycin compounds. In particular, two natural compounds, Epothilone B acid (EPO906) and Epothilone D (KOS‐862), and three semisynthetic derivatives of ebomycin B (BMS247550, BMS310705 and ABJ879).^98^ These compounds have been shown to exert a therapeutic effect on NSCLC, ovarian cancer, and taxane‐resistant tumors.^99^ Among them, BMS247550 was approved for use in the United States by the FDA on October 16, 2007. Thus far, it is the first apomycin analog to have received approval and is considered the most effective antineoplastic drug.^100^

#### 2.3.4 Mitomycin C (MMC)

Mitomycin, also known as mitomycin C, is a cytotoxic antibiotic that was first isolated from *Streptococcus Kaisers* and was approved for use in the USA in 2002.^21^ Mitomycin C is commonly used as a cytotoxic agent against hematological malignancies and other cancers.^101^ It has been used to treat NSCLC since 1984,^102^ and it can be combined with platinum‐based drugs, docetaxel, and other chemotherapies, to improve its response rate.^103^

### 2.4 Marine organisms

#### 2.4.1 Psammaplin A (PSA)

The natural marine chemical product, psammaplin A, was first isolated from *Psammaplysilla* sponges in 1987.^104^ It was synthesized by Hoshino and his colleagues in 1992.^15^ In 1999, psammaplin A was found to exert significant *in vitro* antibacterial activity against Staphylococcus aureus (SA) and methicillin‐resistant *Staphylococcus aureus* (MRSA).^105^ The pharmacological activities of psammaplin A and its derivatives include anticancer, antiviral, and insecticidal effects, as well as the promotion of embryonic development and chemical defensive encryption.^106^ It has also been reported to be an effective inhibitor of two epigenetic enzymes, histone deacetylase, and DNA methyltransferase.^107^

#### 2.4.2 Ecteinascidin‐743 (ET‐743)

Ecteinascidin‐743 is a novel antineoplastic drug extracted from *Ecteinascidia turbinata* found in *the* Caribbean sea *envelope*.^108^ It is a tetrahydroisoquinolone alkaloid, and this novel compound is a groove‐binding guanine specific alkylating agent.^109^ Phase II clinical tests conducted in Europe and America have shown that ET‐743 is a promising antineoplastic drug.^110^ It has been proven to be an effective therapeutic drug against various solid tumor cell lines and human tumor xenografts, including NSCLC, breast cancer, ovarian cancer, prostate cancer, melanoma, and kidney cancer.^111^

#### 2.4.3 Halichondrin B

Halichondrin B was first isolated from an unusual marine Japanese sponge, *Halichondria okadai*, by Hirada and Ishimura in 1985, and then from more commonly used sponges, such as *Axinella*, *Phakellia and Lissodendoryx* species.^112^ Eribulin is a synthetic analog and has been approved by the FDA to treat metastatic breast cancer.^113^ As a tubulin‐binding agent, it can inhibit microtubule dynamics through a different mechanism from that of taxane, and has shown *in vitro* activity on taxane‐resistant cell lines.^114^ In addition, phase III clinical trials have been conducted on MBC, soft tissue sarcoma and NSCLC, bladder/urothelial cancer, pancreatic cancer, neck cancer, salivary gland cancer, prostate cancer, head cancer, and renal insufficiency and ovarian cancer and ovarian cancer‐related gynecological malignancies, while phase II clinical experiments on Eribrin have also been conducted or are being conducted either alone or in combination with other drugs.^115^ The names and sources of these natural compounds are shown in Table 1.

**TABLE 1:** Sources of natural compounds with anticancer activity

Category	Natural compounds	Main Source	References
Plants	Ginsenosides	Ginseng	[11, 12]
	Camptothecin (CPT)	Camptotheca acuminata	[14]
	Curcumin (CUR)	Turmeric	[22, 23]
	β‐elemene (β‐ELE)	Turmeric	[26]
	Gambogic (GA)	Han's Geng Huang resin	[28, 29]
	Tanshinone	Salvia miltiorrhiza	[32, 34]
	Licorice chalcone (LIC)	Licorice	[35, 37, 38, 39, 40, 41]
	Triptolide (TPL)	Tripterygium wilfordii	[42]
	Emodin (ED)	Rhubarb	[44]
	Berberine (BBR)	Coptis chinensis	[48]
	Epigallocatechin gallate (EGCG)	Green tea	[52, 53, 54]
	Resveratrol (RSV)	Grapes, red wine and peanuts	[56]
	Artemisinin	Artemisinin annua	[59]
	Silybin	Silymarin	[62]
	Cinnamon	Cinnamon	[70, 71]
Animals	Astaxanthin (ATX)	Shrimp, salmon, crab, red yeast and other marine animals	[73]
	Melittin	Bee venom	[77, 78]
	Snake venom	Snake	[81, 82, 83]
	Scorpion venoms	Scorpion	[85, 86, 87]
Microorganisms	Doxorubicin (Dox)	Penicillium Streptomyces	[21, 89]
	Staurosporine (STS)	Streptomyces and other actinomycetes	[92]
	Epothilone	myxobacteria and Cysticercus cellulosus	[95]
	Mitomycin C (MMC)	Streptococcus Kaisers	[21]
Marine organisms	Psammaplin A (PSA)	Psammaplysilla sponge	[104]
	Ecteinascidin−743 (ET−743)	Ecteinascidia turbinata	[108]
	Halichondrin B	Halichondria okadai, Axinella, Phakellia, and Lissodendoryx	[112]

## 3 ANTI‐TUMOR MECHANISM

### 3.1 Induction of apoptosis

Apoptosis known as programmed cell death is a series of changes mediated by genes, which has an important effect on maintaining a variety of cell functions, bodies remove aging, and abnormal cells through this process.^116^ The imbalance of apoptosis is an important way for cancer cells to survive, recur and acquire drug resistance, which involves a variety of regulatory mechanisms and signal pathways.^117^, ^118^ This is also an important mechanism for natural products to play an anticancer role.

#### 3.1.1 Inductive ROS

ROS is a natural by‐product of cell metabolism (mainly in the mitochondria). It is composed of hydroxyl groups, superoxide anions, singlet oxygen, and hydrogen peroxide, containing unpaired valence electrons, and therefore high levels of reactivity. Cancer cells in advanced tumors usually show high levels of oxidative stress, indicating that tumor progression requires an increased ROS level and a decrease in the tolerance of cancer cells to ROS.^119^

The Keap1/Nrf2/ARE signal transduction pathway adjusts oxidative stress response, and its high constitutive expression induces chemical resistance and proliferation in many cancer types, including in the development of drug resistance in NSCLC. Ginsenoside Rd (GS‐Rd) can decrease chemical resistance by downregulating the activity of the NRF2.^120^ Triptolide can also significantly inhibit the expression and transcriptional activity of Nrf2, which was found to have increased the chemical sensitivity of cancer cells to antineoplastic drugs *in vitro* and xenograft tumor model systems.^121^

Ginsenoside Rh2 inhibits H1299 cell growth by inducing ER stress‐dependent apoptosis, mediated by ROS.^122^ GA can also inhibit the MAPK/ERK, NF‐κB, and PI3 K/AKT signaling pathways by improving the level of reactive oxygen species ^123^ and induce tumor cell apoptosis and destroy cancer cells through ROS‐induced ER stress.^124^ The inhibitory effect of Tan IIA on the growth of H146 cells may be related to endoplasmic reticulum stress, which can induce ROS release and a reduction in the mitochondrial membrane potential (MMP) caused by an increase in the Bax/Bcl‐2 ratio.^125^

Emodin is most likely to affect cancer prevention/treatment by exerting its effects on mitochondria and acting as a universal crucial effector of cell death, through the reactive oxygen species‐ATM‐p53‐Bax signal pathway.^126^ Su et al. have proven that emodin induces the apoptosis of human NSCLC cells through the reactive oxygen‐dependent mitochondrial signal transduction pathway.^127^ Tanshinone IIA was found to have activated the p53‐independent, ROS‐triggered, and caspase‐dependent mitochondrial apoptotic cell death pathway in A549‐NQO1 and H596‐NQO1 cells, which was characterized by an increase in the Bax/Bcl‐xl ratio, MMP destruction, cytochrome c release, and subsequent caspase activation and PARP‐1 cleavage.^128^ Chiu et al. also found that tanshinone IIA induces the apoptosis of A549 cells by inducing the release of reactive oxygen species and decreasing the MMP.^129^ LCD can induce apoptosis through the accumulation of ROS in lung cancer cells, increasing the loss of MMP and the upregulation or downregulation of mitochondrial‐related proteins, which leads to apoptotic cell death through the mitochondrial (intrinsic) pathway.^41^ To amplify the therapeutic effect of EGCG, magnetic nanoparticles (MNP) encapsulated in bovine serum albumin (BSA) and loaded with EGCG were synthesized to induce apoptosis through the participation of ROS, resulting in the loss of MMP, increase in the expression of Nrf2 and Keap1, regulation of the apoptosis of A549 cells, and induction of EGCG to exert a more substantial anti‐lung cancer effect.^130^ In A549 cells, artemisinin can significantly induce apoptosis through reactive oxygen species‐mediated amplification of the activation loop between caspase‐3, caspase‐8, and caspase‐9.^131^ Besides, Artesunate may play a cytotoxic role in A549 cells and normal HBE cells through ROS‐mediated DNA damage.^60^

#### 3.1.2 Inductive endoplasmic reticulum stress

Endoplasmic reticulum stress (ERS) is a cellular response to protein misfolding, which has a far‐reaching influence on the survival and death of cancer cells. Its activation markers include ATF‐6, CHOP, and ATF‐4, BIP and XBP‐1.^132^ ERS plays a significant role in the pathogenesis of NSCLC. At the same time, the β‐elemene upregulates the expression levels of ER‐related proteins, such as ATF6, PERK, IREα, ATF4, and CHOP, and downregulates the expression of Bcl‐2, which inhibits lung cancer growth and cell viability in a dose‐dependent and time‐dependent manner.^133^ Ginsenoside extract (TGS) can induce NSCLS cell autophagy by activating ERS, mediated by the ATF4‐CHOP‐AKT1‐mTOR axis of NSCLC cells.^132^

Lou et al. studied human lung adenocarcinoma PC9 cell lines and transplanted tumor models into nude mice and found that tanshinone showed sound anti‐lung cancer effects. Simultaneously, its mechanism of action was related to the promotion of apoptosis induced by ERS and the activation of the IRE1 α/caspase‐12 apoptosis pathway.^134^ The inhibitory effect of Tan IIA on the proliferation of H146 cells may be related to ERS, which is caused by the release of Ca^2+^ and the increased expression of GADD153, while the decrease in the expression of Bcl‐2 leads to an increase of the Bax/Bcl‐2 ratio, which in turn leads to a decrease in MMP and increased caspase‐3 expression.^125^

**FIGURE 1:**
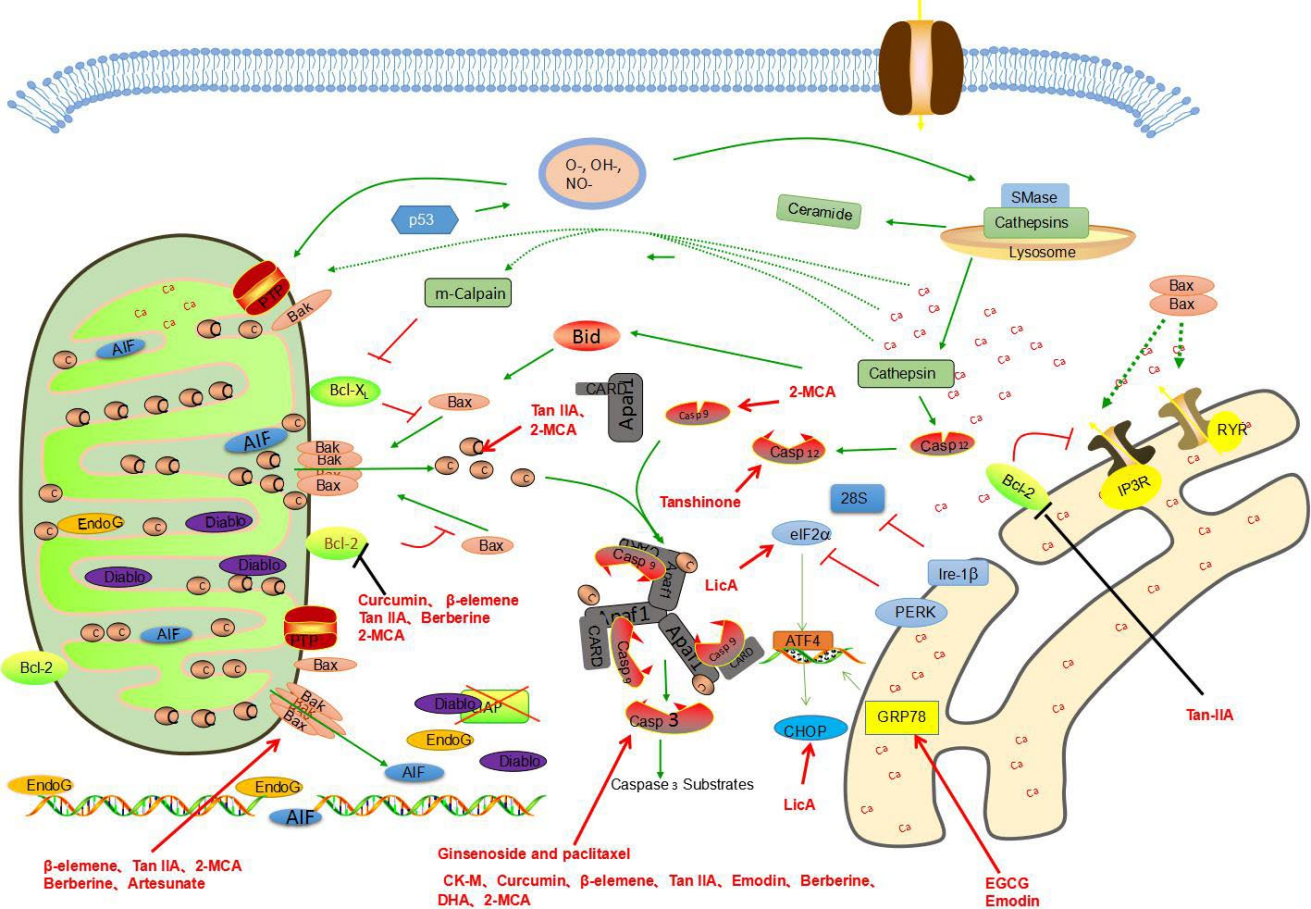
Natural extracts can induce apoptosis through ERS pathway and mitochondrial pathway. In this and below figures, the red thick line arrow indicates that the compound can increase the expression or activity of the corresponding protein, and the thick black line indicates that the compound can reduce the expression of the corresponding protein or inhibit the activity of the protein

LicA increases the expression levels of ERS related proteins, such as p‐EIF2 α and ATF4, to inhibit the proliferation of lung cancer cells.^135^ LicA also significantly promotes the expression of the tumor suppressor, miR‐144‐3p, which upregulates the ER stress response protein, CHOP, and induces lung cancer cell apoptosis by downregulating the expression of the nuclear factor E2‐related protein (Nrf2).^136^ CHOP, which is known as a DNA damage‐inducing and growth arrest gene, is one of the most critical components of the ER stress network and its activation is essential for LicA‐induced apoptosis, cell viability, and autophagy.^39^ LicA treatment induces ER stress activation, which leads to apoptotic cell death and exerts an anti‐tumor effect that significantly decreases the level of cisplatin‐induced renal damage but does not affect its anticancer effect.^135^ GRP78 is the molecular chaperone of the ER that contributes to the correct folding of newborn peptides. When unfolded proteins accumulate, GRP78 triggers the unfolded protein response (UPR), which activates transcription factors such as XBP1 and CHOP, to restore stressed cell homeostasis. The increased expression of GRP78 and mild UPR may be present in cancer cells that promote cell survival and prevent apoptosis. Exposure to EGCG further increases GRP78 expression in the ER and induces XBP1, CHOP, and EDEM expression spliced by ATF4. It also decreases the expression of GRP78 on the cell surface and increases the activity of caspase‐8 and caspase‐3.^137^ GRP78 and CHOP expression can also be detected in lung cancer cells treated with emodin, indicating that emodin can induce the apoptosis of lung cancer cells mediated by ERS.^138^ (Figure 1).

#### 3.1.3 Induction of apoptosis through the mitochondrial pathway

Mitochondria play a significant role in the physiology of cancer cells. Most tumor cells are resistant to apoptosis and can be regarded as the primary regulators of cell life and death.^117^ The most decisive event induced by the mitochondrial pathway during apoptosis is mitochondrial outer membrane permeability (MOMP), with MOMP being highly regulated by members of the BCL‐2 protein family. This family can be categorized as pro‐apoptotic proteins (BAK and BAX), pro‐apoptotic BH3 protein (BIM, Noxa, BMF, BIK, PUMA, BID, HRK, and BAD) and anti‐apoptotic BCL‐2 protein (BCL‐xL, BCL‐BA1, BCL‐2, MCL‐1, and BCL‐w). After MOMP, mitochondrial intermembrane space proteins (especially cytochrome c) are released into the cytoplasm, activating cystatin.^139^

The combination of ginsenoside and paclitaxel was found to have upregulated the expression of caspase‐3 protein and downregulated the ratio of Bcl‐2/Bax, which significantly increased the apoptosis‐inducing effect on A549 cells.^140^ CK‐M exerts a good tumor targeting effect within 24 hours and maybe a suitable delivery agent to enhance the solubility and anti‐tumor effect of CK. TPGS/PEG‐PCL mixed micelle (CK‐M) promotes tumor cell apoptosis and inhibits tumor cell metastasis, invasion, and efflux by regulating levels of Bax, Bcl‐2, matrix metalloproteinase‐2, P‐glycoprotein, caspase‐3, and caspase‐2.^141^ The combination of curcumin and cisplatin significantly inhibited A549/DDP cell proliferation, reversed DDP resistance, and induced apoptosis by activating caspase‐3, and promoting HIF‐1α degradation.^142^ In addition, curcumin reduces Bcl‐2 levels through ubiquitin‐proteasome degradation, making NSCLC cells sensitive to apoptosis.^143^ β‐elemene increased the expression of Bax, decreased the expression of Bcl‐2 and induced PARP cleavage, and enhanced apoptosis induced by β‐elemene.^144^ The combination of β‐ELE and cisplatin decreased the protein levels of procaspase‐3 and Bcl‐2 in A549/DDP cells and increased the protein expression of caspase‐3, cytochrome c, and Bad in A549/DDP cell lines, indicating that β‐ELE reversed the drug resistance of A549/DDP cells by inducing apoptosis.^145^

Tan IIA can also induce JNK signal activation and trigger cascade apoptosis mediated by cytochrome c release.^146^ Tan IIA combined with cyclophosphamide (CTX) can regulate Bcl‐2 and Bax expressions in lung cancer tissues of Lewis mice, inhibit the neovascularization of tumor tissue, enhance immune function, and exert evident anti‐tumor activity.^147^ Li et al. found that emodin can inhibit the proliferation of the lung adenocarcinoma cell line, Anip‐973 by activating caspase‐3 induced apoptosis and arresting the cell cycle.^148^ It also enhanced the apoptosis of A549 cells induced by PTX and the anti‐tumor effect on A549 xenografts by increasing the expression of Bax and active cystatin‐3 and decreasing levels of p‐Akt, Bcl‐2, and p‐ERK, while there were no apparent side effects in vivo.^44^ A549 cells expressing wild‐type p53 and H1299 cells with p53 deficiency could inhibit cell proliferation and increase the apoptosis rate when A549 cells were treated with berberine. A549 cells were more sensitive to berberine‐induced cytotoxicity. Apoptosis of A549 and H1299 cells induced by berberine was related to the destruction of mitochondrial membrane potential, a decrease of Bcl‐2 and Bcl‐xl levels, and the increase of Bax and Bak levels, as well as activation of caspase‐3.^149^

Artesunate can induce apoptosis through the Bak‐mediated caspase‐independent intrinsic pathway in human NSCLC cells.^150^ Dihydroartemisinin (DHA), a semisynthetic derivative of artemisinin extracted from the Chinese herbal medicine, *Artemisia annua*, induced apoptosis of SPC‐A‐1 cells by downregulating the expression of surviving at mRNA and protein levels, but did not affect caspase‐4.^151^ It was also able to inhibit the proliferation and induce the apoptosis of ASTC‐a‐1 cells through the caspase‐3 dependent mitochondrial death pathway.^152^ Methoxycinnamaldehyde (2‐MCA), a component of cinnamon bark, was also able to induce apoptosis and inhibit proliferation, which was characterized by the upregulation of Bax and Bak genes and pro‐apoptotic and downregulation of Bcl‐2, anti‐apoptotic and Bcl‐XL genes, the release of caspase‐3, loss of MMP, cytochrome c and caspase‐9 and their activation.^153^ (Figure 1).

### 3.2 Induction of autophagy

Autophagy is a biological process that is stimulated in response to various stresses (including hunger, reactive oxygen species (ROS), hypoxia, and DNA damage), in which membrane receptors receive signals transmitted to the cell,^14^ and maintain balance in the body by capturing and degrading damaged proteins and organelles, which in turn is considered to promote an inhibitory effect on cancer cells.^154^

There are two types of autophagy: the first type, known as protective autophagy, protects against apoptosis, while the other is autophagic cell death, which induces the death of cancer cells. The GA‐induced autophagy of NCI‐H441 cells belongs to the latter type of autophagy and is mediated by ROS production.^119^ Compared with tanshinone IIA, total tanshinone (TDT) showed more potent cytotoxic effects on 95D lung cancer cells, and apoptosis and protective autophagy induced by TDT were also mediated by an increase in intracellular ROS production.^155^ When A549 cells were treated with cryptotanshinone (CTS), autophagic vesicles were found to have accumulated and the expression of LC3 protein and autophagosomes increased, proving that cryptotanshinone could exert its anti‐tumor effect by promoting autophagy.^156^ Curcumin can induce autophagy by controlling hST8SiaI gene expression, related to the autophagy of A549 cells through the AMPK signaling pathway.^157^ Wild‐type p53 is a tumor suppressor protein that is important for cancer prevention. Emodin can target the accumulation of p53 protein in A549 cells and increase autophagy.^158^ In addition, resveratrol is an activator of SIRT1 that induces protective autophagy in NSCLC by activating p38‐MAPK and inhibiting the Akt/mTOR pathway ^159^ to overcome gefitinib resistance through autophagy and aging.^160^

**FIGURE 2:**
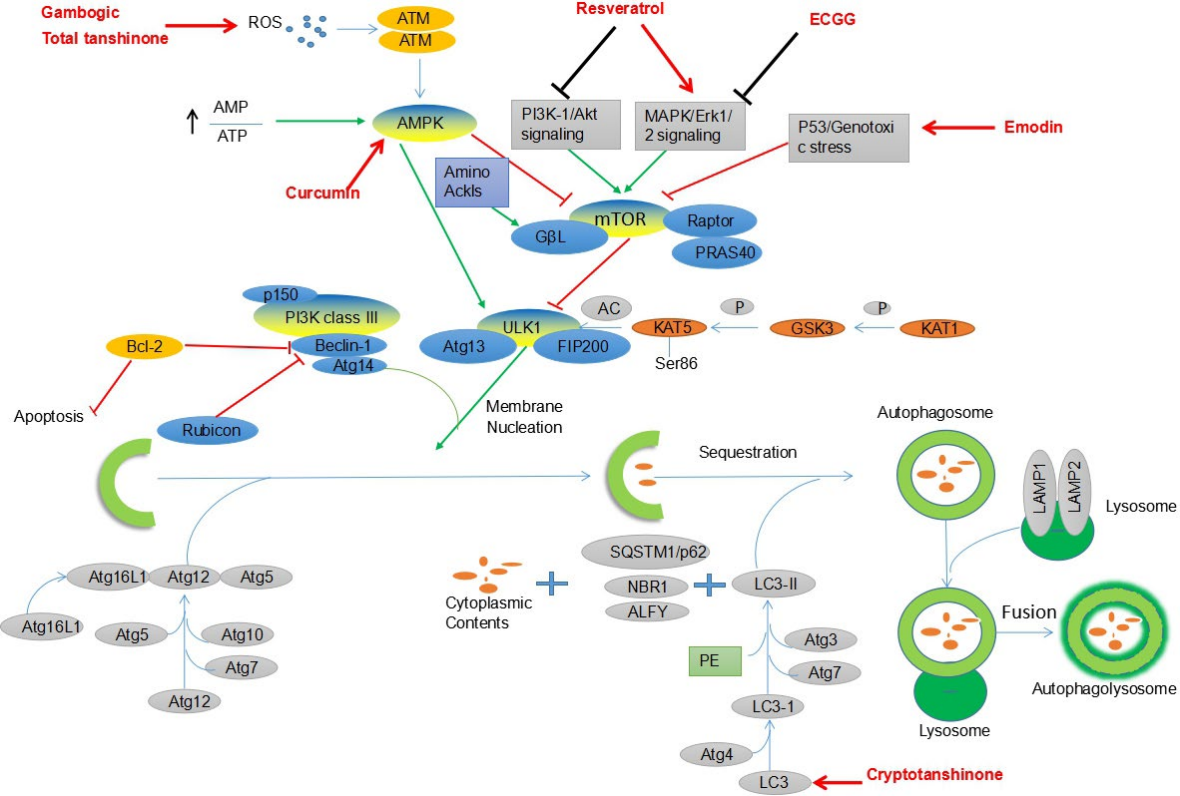
Natural extracts exert inhibitory effects on lung cancer by inducing autophagy

Many studies have shown that autophagy can enhance tumor resistance to chemotherapy, targeted therapy and radiotherapy.^161^ The inhibition of autophagy heightens the level of DNA damage induced by CPT in lung cancer cell lines, indicating that autophagy exerts a protective effect on lung cancer cells treated with CPT, and the combination of CPT and specific autophagy inhibitors can be considered as a promising therapeutic method for lung cancer in the future.^14^ The inhibition of autophagy induced by Rg3 can increase the therapeutic response of etinib‐sensitive and etinib‐resistant NSCLC cells with EGFR‐activated mutations.^162^ EGCG overcomes gefitinib (Gef) resistance by targeting ERK phosphorylation in NSCLC to inhibit autophagy and enhance cell death.^163^ (Figure 2).

### 3.3 Inhibition of the PI3 K/Akt signaling pathway

It is well known that the PI3 K/Akt pathway is one of the most vital carcinogenic pathways in almost all cancers. The PI3 K/Akt signaling pathway has an essential effect on regulating cell survival, growth, apoptosis, and autophagy.^164^ Activated Akt regulates cell differentiation, proliferation, apoptosis, and migration by inhibiting a series of downstream substrates, such as caspase‐9, GSK23, NF‐κB, and Bad.^165^ Therefore, the PI3 K‐AKT pathway seems to be one of the most promising targets for anticancer drugs.

Rad51 activity is related to tumorigenesis or tumor progression and may be a useful prognostic marker in NSCLC. Its high expression indicates a significant decrease in the survival rate. Astaxanthin downregulates Rad51 expression by inactivating AKT to enhance the mitomycin C‐induced cytotoxicity of human NSCLC cells.^74^ PTEN negatively regulates the Akt signal pathway by degenerating PIP3 phosphate into PIP2. The PTEN gene is often mutated in many different types of cancers. A study observed that CTN treatment increased the upregulation of PTEN, while PTEN degraded PIP3, resulting in a decrease of Akt phosphorylation of Ser 473 and Thr 308. Akt inactivation reduces the degradation of p53, which ultimately leads to the increased expression of p21 and p53, and inhibition of tumor progression, indicating that tanshinone induces apoptosis through the mitochondrial apoptosis pathway and inhibition of the PTEN‐mediated PI3 K/Akt pathway for effectively inhibiting tumor growth.^164^ PESV (peptides from BmK scorpion venom) can also increase the expression of PTEN in cultured A549 cells.^86^ Epothilone B may enhance the apoptosis of human cancer cells induced by the Bcl‐2 antagonist, ABT‐737, through the PI3 K/AKT/mTOR signal pathway.^99^ EGCG can inhibit the proliferation and induce apoptosis of H1299 cells, which is relevant to the inhibition of the activation of the PI3 K/Akt pathway and downregulation of the expressions of p‐PI3 K and p‐Akt in lung cancer cells.^165^

Metastasis is a multi‐step process that involves the overexpression of proteolytic enzymes, such as urokinase‐type plasminogen activator (u‐PA) and matrix metalloproteinase (MMP), MMP or u‐PA gene expression, mainly at the transcriptional level (through the MAPK or PI3 K‐Akt pathway through AP‐1 or NF‐κB) and post‐transcriptional level, and it is regulated or inhibited by its activator at the protein level. Silybin can prevent Akt phosphorylation from inhibiting the PI3 K‐Akt signaling pathway and inhibiting lung cancer cell invasion.^166^ In addition, Rg3 may also inhibit the apoptosis of lung cancer cells by inhibiting the PI3 K/Akt pathway.^167^

Extensive studies have shown that MDR cells can resist apoptosis induced by anticancer drugs by upregulating survival signaling pathways (including PI3 K and ERK1) or inhibiting antiproliferative signaling pathways, including the p38 MAPK. Triptolide promotes the apoptosis and cell cycle arrest of drug‐resistant A549/Taxol cells by regulating the MAPK and PI3 K/Akt signaling pathways.^168^ The combination of triptolide and hydroxycamptothecin induces the apoptosis of human lung cancer cells by increasing PP2A activity, activating p38, ERK, and MAPK cascade reactions and by inhibiting the Akt survival pathway through mechanisms that involve PP2A activation, which synergistically enhances the effect of triptolide on A549 lung adenocarcinoma cells.^169^

GA and cisplatin (CDDP) produced a synergistic growth inhibition effect on NCI‐H460 and A549 cells. GA treatment decreased the activation of Akt, mTOR, and S6, which may target autophagy‐dependent cell death by activating the Akt/mTOR signaling pathway.^170^ The combination of tanshinone IIA and cisplatin at a ratio of 20:1 can impair cell invasion and migration, block the cell cycle at the S phase, and induce the apoptosis of A549 and PC9 cells in a synergistic manner, probably due to the downregulation of the expression of the p‐Akt and p‐PI3 K proteins, which affect the PI3 K/Akt signaling pathway, as a result of the activity of tanshinone IIA.^171^ The synergistic anti‐tumor activity of curcumin and carboplatin is due to the inhibition of Akt phosphorylation that inhibits the Akt/IKKα pathway and enhancement of ERK1/2 activity to inhibit NF‐κB, allowing carboplatin to act as a chemical sensitizer.^172^ Wang et al. demonstrated that Tan IIA enhanced NSCLC cell sensitivity to gefitinib by downregulating the VEGFR2/Akt pathway (downregulating the phosphorylation levels of VEGFR2 and Akt) in vivo and in vitro.^173^

**FIGURE 3:**
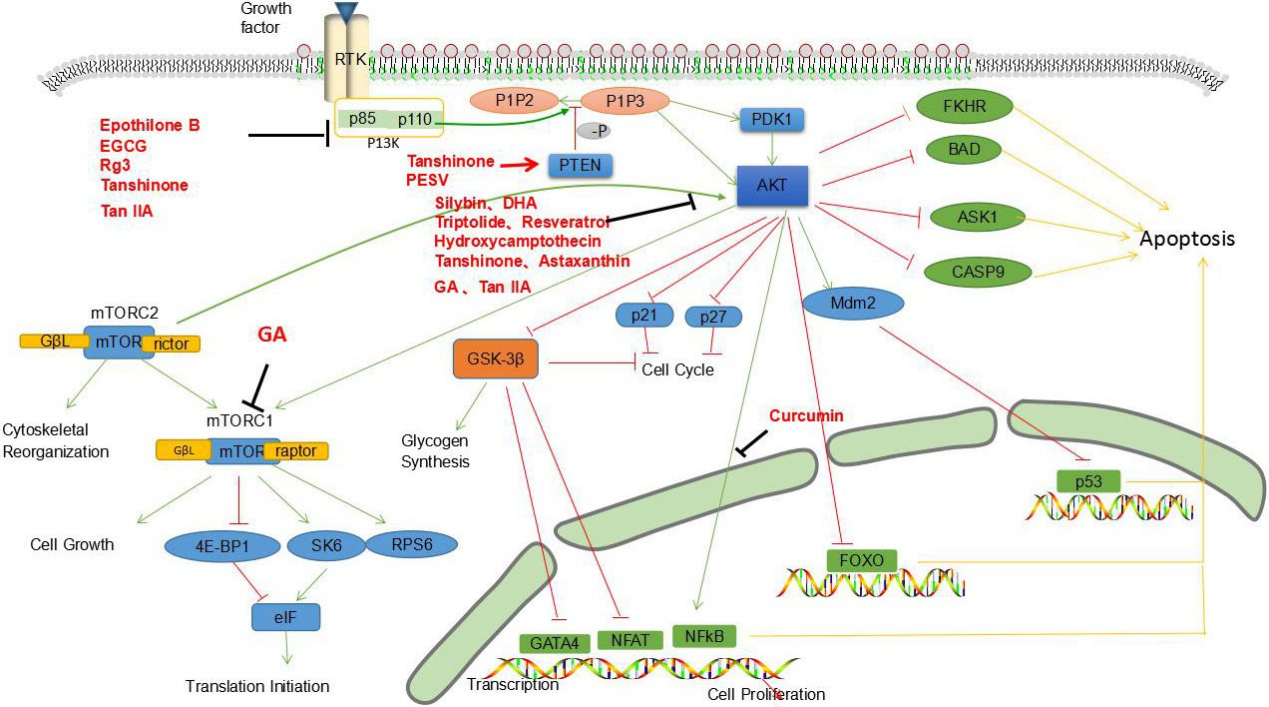
Natural extracts exert inhibitory effects on PI3 K/Akt signaling pathway

XRCC1 is a major scaffolding protein involved in base excision repair. It is regulated by ERK1/2 and AKT signaling and is significantly involved in lung cancer development. Resveratrol enhances the etoposide‐induced cytotoxicity of human NSCLC cells by downregulating ERK1/2 and AKT‐mediated XRCC1 protein expression.^174^ In NCI‐H1975 cells treated with DHA and gefitinib, the expression of pMIT and p‐STAT3 was significantly downregulated, indicating that both of them synergistically inhibit the growth and promote the apoptosis of NSCLC cells through the Akt/mTOR/STAT3 pathway.^175^ (Figure 3).

### 3.4 Inhibition of the NF‐κB signaling pathway

There is considerable evidence that NF‐κB is constitutively activated in many types of solid tumors, including cervical cancer, pancreatic cancer, lung cancer, breast cancer, and prostate cancer.^176^ NF‐κB exerts its role by activating various downstream signal transduction cascades, such as the TNF‐α, BCL‐2, and STAT 3 pathways. Previous studies have shown that increased expression of NF‐κB can be detected in NSCLC tissues and acts as a tumor promoter in NSCLC. In addition, the overexpression of NF‐κB is related to cancer cell metastasis and the poor prognosis of patients with NSCLC.^177^

Epithelial‐mesenchymal transformation (EMT) is an essential factor during the metastasis of bronchogenic carcinoma. Rg3 inhibits EMT and the invasive ability of lung cancer by downregulating EGFR inactivation mediated by FUT4 and blocking the MAPK and NF‐κB signaling pathways.^178^ Saponin Rg3 + cisplatin can inhibit hypoxia‐induced EMT and cancer cell stemness due to the inactivation of the NF‐κB signaling pathway caused by Rg3.^13^ Extracellular ATP performs many significant functions by activating P2 receptors on the cell surface. Emodin inhibits ATP‐induced EMT, migration, and proliferation by inhibiting the P2Y receptor‐mediated increase of Ca^2+^ and NF‐κB signal transduction in A549 cells.^179^ GA inhibits NF‐κB signal transduction, in turn inhibiting the EMT process induced by TGF‐β1 through the inhibition of the expression of TWIST1, which leads to inhibition of lung growth and metastasis of A549 cells.^180^

Programmed death ligand 1 (PD‐L1) is highly expressed on the surface of a variety of human cancer cells, such as malignant melanoma, NSCLC, hepatocellular carcinoma, and ovarian cancer. It has been confirmed that it is involved in the chemotherapeutic resistance of NSCLC. PD‐L1 is a new target for the treatment of lung cancer cells. Rk1 inhibits PD‐L1 expression and promotes apoptosis by inhibiting NF‐κ B signal transduction. Thus, it inhibits the proliferation of A549 and PC9 cells.^181^ Rg3 can inhibit the growth of A549/DDP cells and reduce their resistance to cisplatin by preventing the expression of NF‐κB from decreasing the expression of PD‐L1 induced by chemical resistance and restoring the cytotoxicity of T cells toward cancer cells.^182^

Low concentrations of triptolide may bind to high‐affinity targets to activate p38α and ERK1/2, and block the activation of NF‐κB induced by TNF‐α and IL‐1 β through p53.^183^ Luteinic acid and its analogs inhibit IKKβ kinase activity by inhibiting the activation of the TNFα/NF‐κB pathway, which in turn induces apoptosis in A549 and U251 cells.^184^ DHA can inhibit lung cancer cell metastasis by inhibiting the NF‐κB/GLUT1 axis.^185^ The inhibitory effect of silybin on lung tumor growth can also be achieved through the regulation of macrophage‐associated cytokines, NF‐κB, as well as signal transduction and transcriptional activators on angiogenesis.^186^ BV can induce apoptosis of A549 and NCI‐H460 cells by increasing the expression of death receptor 3 (DR3) and inhibiting the NF‐κB pathway. BV combined with the TNF‐like weak apoptosis inducer, docetaxel, and cisplatin can synergistically inhibit the growth of A549 and NCI‐H460 cells further to downregulate the activity of NF‐κB.^187^

**FIGURE 4:**
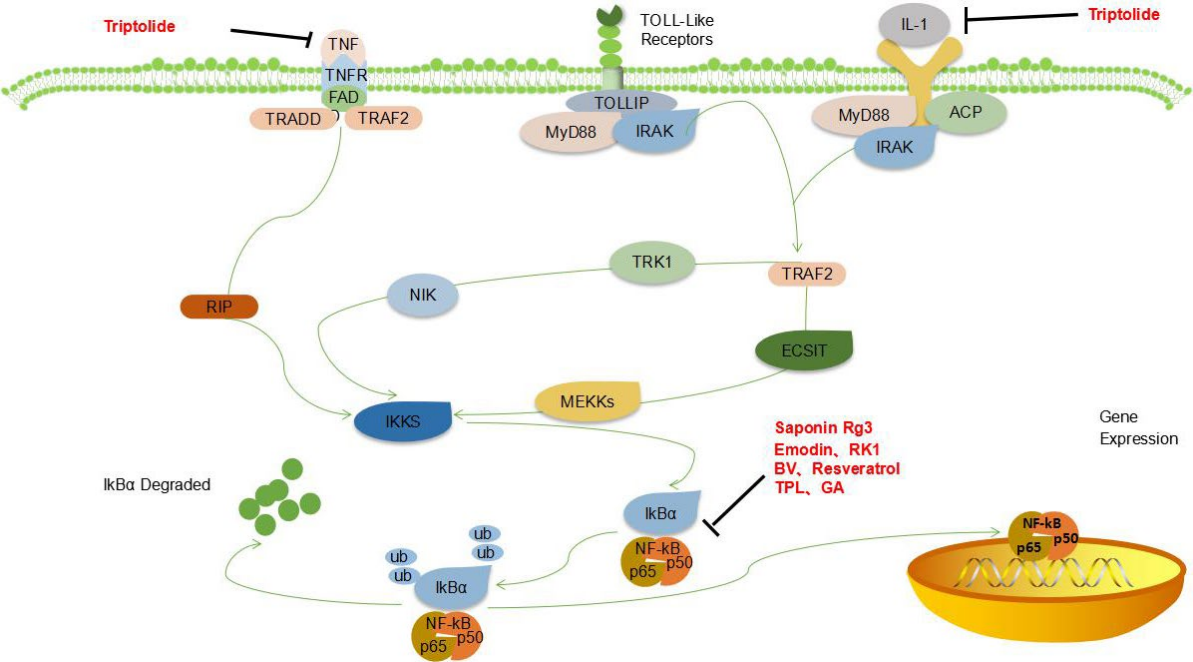
Natural extracts block NF‐κB signaling pathway

The resveratrol targeting NF‐κB (p65) pathway can decrease TRAIL drug resistance, sanitizing lung cancer cells sensitive to TRAIL, and allowing the induction of TRAIL‐mediated apoptosis.^60^ TPL also inhibits the activation of NF‐κB by blocking the transactivation of p65, sanitizing A549, and NCI‐H1299 cells to apoptosis induced by TRAIL.^188^ It can also reverse the paclitaxel resistance of lung cancer by inhibiting the NF‐κB signaling pathway and regulating the transcription and expression of drug resistance genes.^189^ Ginsenoside Rg3 can inhibit the activation of NF‐κB, the phosphorylation of IκB protein and the expression of NF‐κB regulated gene products (promotes apoptosis, cyclin‐D1, cyclin‐2, cyclooxygenase‐2, and VEGF) by targeting the NF‐κB pathway, sensitizing human NSCLC cells to γ radiation.^190^ The synergistic anti‐tumor activity of curcumin and carboplatin is due to the inhibition of the Akt/IKKα pathway and the enhancement of ERK1/2 activity that inhibits NF‐κB for carboplatin to act as a chemical sensitizer.^172^ GA and ADM (Adriamycin) exert a potent anti‐tumor effect on the A549 xenograft model by inhibiting P‐glycoprotein and NF‐κB, attenuating ADM‐induced cardiotoxicity, and sensitizing lung cancer cells to ADM.^191^ (Figure 4).

### 3.5 The arrest of the cell cycle

The regular process of cell division occurs through the cell cycle, which is a sequence of highly ordered steps, which are usually divided into G0/G1, S, G2, and M phases. These steps are regulated at the molecular level by several regulators, including cyclin‐dependent kinase 1 (CDK1), cyclin‐D, and CDK inhibitor (CKI).^192^, ^193^ In recent years, studies have found that normal cells proliferate only in response to specific mitotic signals and growth stimuli, while cancer cells proliferate in an uncontrolled manner. In addition, almost all molecular types involved in the regulation of cell proliferation are involved in malignant transformation. Therefore, cancer can be regarded as a cell cycle disease.^194^

Myc expression is strictly controlled by the availability of mitogens in normal cells but is usually misregulated or elevated in tumor cells.^195^ Emodin can downregulate the C‐myc gene expression to induce cell cycle arrest and induce growth inhibition of A549 cells.^47^ TPL inhibits lung cancer cell proliferation and tumor growth through apoptosis and cell cycle arrest induced by nucleolus decomposition and rRNA synthesis inhibition (possibly through the ribosomal‐RPL23‐MDM2‐p53 signaling pathway).^196^ BBR inhibits AP‐1 signaling pathway activity and reduces the binding of transcription factors to the CCND1 AP‐1 motifs, which is an essential mechanism for the action of berberine as a cyclin‐D1 regulator against human lung giant cell carcinoma.^197^ Low concentrations of ART inhibit mRNA levels of cyclin‐related genes, including CDK2, CDK1, cyclin‐D1, CDK6, cyclin‐B1, and cyclin‐A2, which lead to cell cycle arrest of NSCLC cells.^198^ Silybin inhibits human NSCLC cell growth by regulating the expression and function of key cell cycle regulators, reducing the protein levels of G1‐related CDK^2^, ^4^, ^6^ and their corresponding cyclins‐D1 and D3, and inducing arrest of the cell cycle at G1.^199^ The combination of silybin and indole‐3‐methanol (I3C) also enhanced the inhibitory effect of lung adenocarcinoma in A/J mice,^65^ mainly by inhibiting the inflammatory drive by regulating key cell cycle regulatory factors and reducing the level of cyclin‐D1 and related proteins.^200^ The Rb gene plays an essential role in regulating tumor cell proliferation, growth, and apoptosis. The Rb gene was found to be activated by a low concentration of mitomycin C enhanced the inhibition of proliferation, significantly increased the number of cells at the G1/G0 phase and decreased the number of cells at the S and G2/M phases, indicating that the inhibitory effect of mitomycin C on A549 cells is induced mainly through cell cycle arrest.^101^

Ginsenoside Rh2 can activate the JNK/MAPK signaling pathway, increase the transcriptional and phosphorylation activity of transcription factors(ATF2 and AP‐1), reduce the expression of transcription factors(C‐myc and E2F1), and affect the expression of CDK4 and cyclin‐D1, which are the critical regulators of G1/S cyclin‐dependent kinases.^201^ Saha also found that curcumin can induce the expression of cyclin‐dependent kinase inhibitors, p27 and p21, and inhibit the expression of CDK2, CDK4, cyclin‐D1, CDK6, and other genes, to block the cell cycle in the G1/S phase and inhibit the growth of PC‐9 cells.^202^ Protein arginine methyltransferase 5 (PRMT5) is associated with the development of many types of cancers and tumors, especially lung cancer. PRMT5 promotes the apoptosis of lung cancer cells through Akt/Gsk3β signaling induced by resveratrol. Further studies have shown that the inhibition or downregulation of PRMT5 further reduces the phosphorylation of Akt/GSK3β and the expressions of cyclins‐D1 and E1 among the downstream target cells treated with resveratrol.^203^ This induces cell cycle arrest at the G0 / G1 phase in lung cancer cells.^204^ UCN‐01 (7‐hydroxystaurosporine) can inhibit CDKs and inhibit cell cycle progression of A549 cells from G1 to the S phase.^205^

GA was found to have induced the apoptosis of A549 cells in a time and dose‐dependent manner arrested the cells at the G0/G1 phase in vitro, and downregulated the mRNA expression of cyclin‐D1 and COX‐2, suggesting that GA could inhibit the proliferation of tumor cells through apoptosis induction and cell cycle arrest.^206^ Both tanshinone nano‐emulsion and extract could penetrate the cytoplasm through endocytosis, which could induce the upregulation of p53 and p21 and downregulation of CDK2, cyclin‐E1, and cyclin‐D1. At the same time, the cell cycle was arrested at the G0/G1 phase, and tanshinone nano‐emulsion was found to have inhibited the proliferation of A549 cells more effectively than tanshinone extract.^207^ Dihydroartemisinin exerts apparent anticancer activity on A549 cells, which is related to G0 and G1 phase arrest.^208^ DHA treatment of A549 cells led to cell cycle arrest at the G1 phase, which was related to the downregulation of PCNA and cyclin‐D1 at mRNA and protein levels.^209^ DHA induces potent cytotoxicity and radiosensitivity on GLC‐82 cells. The mechanism of action may proceed by preventing the growth of GLC‐82 cells at the G0/G1 phase, reducing the proportion of cells at the S phase, restoring p53 function, reducing the expression of Bcl‐2 protein, and inducing GLC apoptosis.^210^ Melittin was found to have induced G1 cell cycle arrest of Chago‐K1 human bronchial cancer cells.^211^

**FIGURE 5:**
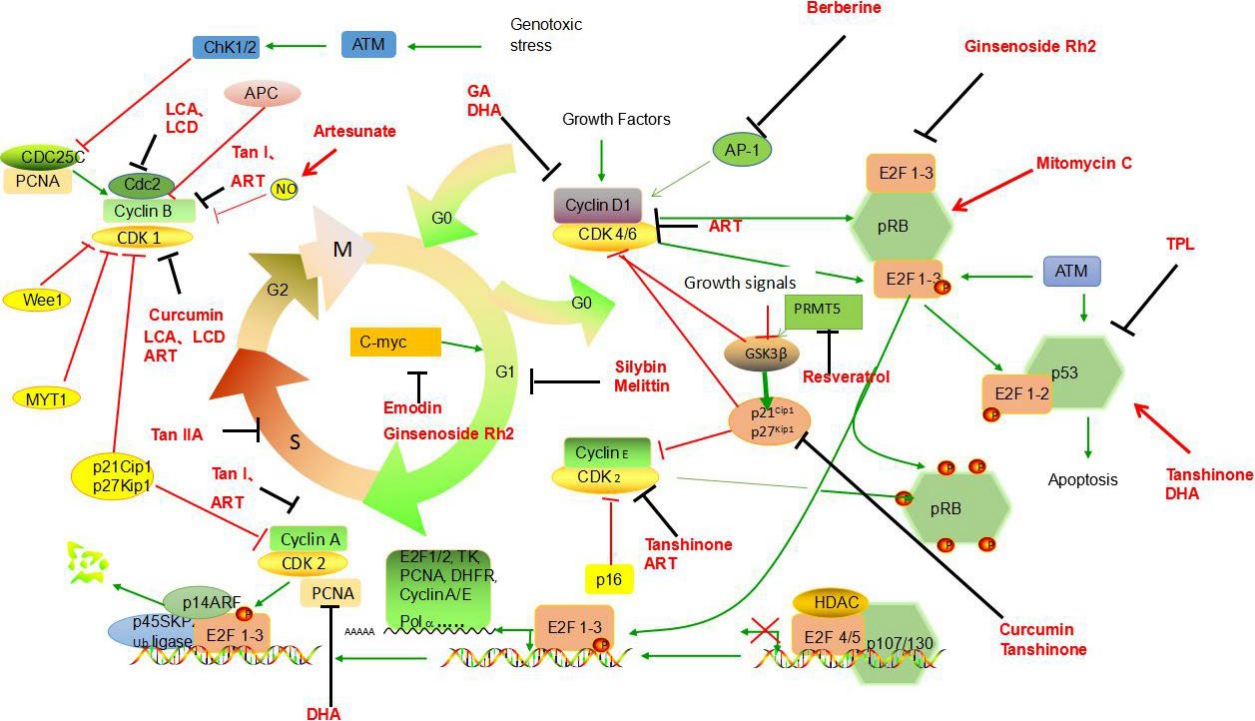
Natural extracts can induce cell cycle arrest in lung cancer cells

Tan IIA may block the VEGF/VEGFR signaling pathway, indirectly induce S‐phase cell cycle arrest and apoptosis and inhibit the downstream signaling pathway, and subsequently upregulate the expression of apoptosis‐promoting genes and downregulate the expression of anti‐apoptosis genes, and finally inhibit the growth and proliferation of A549 cells.^212^ Tan1 inhibits the growth and angiogenesis of lung carcinoma cells in a dose‐dependent manner by inhibiting the expression of cyclin‐A, VEGF, and cyclin‐B proteins, inducing a stronger effect than Tan2. This anti‐tumor effect may slow down the progression of cells through the S and G2/M phases of the cell cycle.^213^ When lung cancer cells were treated with a nano‐emulsion of curcumin extract, it was found that H460 cells were more prone to apoptosis than A549 cells and that the cell cycle remained in the G2/M phase, accompanied by a dose‐dependent decrease in CDK1 expression.^214^ LCA and LCD significantly decreased the expression of cyclins‐B1 and cdc2 in lung cancer cells, and their complexes participated in the G2/M phase transition, which is that G2/M cell cycle arrest could inhibit the proliferation of lung cancer cells.^36^, ^41^ Artesunate can increase the production of NO to induce cell cycle arrest at the G2/M phase, which is relevant to the downregulation of cyclin‐B1 mRNA expression and improves the radiosensitivity of human NSCLC A549 cells.^215^ ET‐743 induces the characteristics of medium cell line‐dependent radiosensitization in A549 cells, and the radiosensitization may be caused by G2/M phase arrest.^111^ (Figure 5).

### 3.6 Regulation of epigenetics

Few studies have shown that epigenetics plays a crucial part in the development of lung cancer. Epigenetic mechanisms, including histone modification, DNA methylation, chromatin tissue, and non‐coding RNA, are hereditary and reversible. Many genes are silenced or uncontrolled during the carcinogenesis of lung cancer.^216^

MicroRNAs (miRNAs) are non‐coding RNA of a length of 18–25 nucleotides, which are expressed in all cells. Their abnormal expression leads to an abnormal protein function, which leads to NSCLC tumorigenesis. Tumor tissue factor (TF) contributes to cancer metastasis in some NSCLC patients. BBR inhibits NSCLC cell growth and promotes apoptosis through the miR‐19a/TF/MAPK signaling pathway.^217^ Mir‐491 antisense oligodeoxynucleotides can inhibit hypoxia‐induced migration, while Rh2 can exert anti‐metastatic activity on the hypoxic tumor microenvironment of lung adenocarcinoma cells by increasing the expression of mir‐491.^218^ MiR‐301a‐3p is a miRNA that has been suggested to exert carcinogenic effects on many types of cancers. β‐elemene may weaken the Warburg effect in NSCLC cells by mediating the miR‐301a‐3p/AMPKα axis by inhibiting the expression of miR‐301a‐3p in NSCLC cells and increasing the expression of AMPKα.^219^ In addition, β‐elemene increases IGFBP1 gene expression through Stat3 inactivation and then produces a mutual effect between miRNA155‐5p and FOXO3a, which leads to the inhibition of the growth of lung cancer cells.^220^ Melittin activates caspase‐2 (CASP2) to induce lung carcinoma cell apoptosis by inhibiting miR‐183 expression.^221^

AURKA is a carcinogenic gene that encodes for serine‐threonine kinase and regulates mitosis in mammalian cells. Tanshinone can inhibit AURKA, by upregulating the expression of miR‐32 and other related miRNAs to inhibit NSCLC.^222^ LICA was found to have reversed the expression of ectopic miRNAs induced by NNK, including miR‐328‐3p, let‐7d‐3p, miR‐29c‐3p, miR‐20a‐5p, and miR‐144‐3p, to stimulate chemoprophylaxis in vivo and in vitro.^223^ Triptolide decreases proliferation and enhances NSCLC cell apoptosis by targeting miR‐21 to enhance the expression of phosphatase and tensin homolog protein (PTEN).^224^ HOTAIR is an important carcinogenic lncRNA that is involved in invasion and tumorigenesis, while miR‐34a‐5p plays a role in tumor suppression. The combination of BBR and gefitinib may control the growth and metastasis of NSCLC through miR‐34a‐5p and HOTAIR‐mediated EMT inhibition.^225^

Zhang et al. first proved that curcumin exerts anticancer effects on A549/DDP multidrug‐resistant cells, which changes miRNA expression, primarily by reducing the expression of miR‐186.^226^ Curcumin can improve the sensitivity of paclitaxel‐resistant NSCLC cells to paclitaxel through microRNA‐30c‐mediated MTA1 reduction.^227^ Cancer stem cells (CSCs) are considered the leading cause of tumor metastasis, recurrence, and chemotherapeutic resistance. In serum samples of NSCLC patients, hsa‐mir‐485‐5p expression was found to have decreased, while RXRα (a nuclear receptor) expression was found to have increased in NSCLC. The upregulation or activation of RXRα enhanced the CSC‐like characteristics of NSCLC cells. EGCG can inhibit CSC‐like characteristics by regulating the hsa‐mir‐485‐5p/RXRα axis.^228^ The overexpression of miR‐485 could reduce the stemness of A549/DDP cells, while EGCG could inhibit stemness by increasing the expression of miR‐485 in A549/CDDP cells in a dose‐dependent manner.^229^ The miRNA spectrum analysis shows that the upregulation of Myb by EGCG can be realized by the downregulation of miRNA, mmu‐miR‐449c‐5p induced by EGCG.^230^ Using next‐generation sequencing technology, KEGG analysis, and the PANTHER pathway showed that the MAPK pathway is the most effective targeting pathway for EGCG‐regulated microRNAs.^55^

As an oncogene, the novel long non‐coding RNA, AK001796, is concerned with cell growth inhibition induced by resveratrol in lung cancer. Microarray analysis has shown that AK001796 was the most apparent long non‐coding RNA (lncRNA), and that it is overexpressed in lung cancer cells, but that its expression was downregulated in lung cancer cells treated with resveratrol, and that the decrease of the lncRNA AK001796 level may weaken the inhibitory effect of resveratrol on cell proliferation.^231^ Silybin can also reverse the drug resistance of human SCLC cells,^232^ downregulate the expression of miR‐21, and promote the re‐expression of miR‐200c in erlotinib refractory tumors, as well as inhibit EMT‐driven erlotinib resistance.^233^

Histone deacetylases (HDAC) are epigenetic enzymes that control gene expression by inhibiting histone deacetylation transcription. BBR inhibits HDAC‐mediated epigenetic reprogramming, which may be a key mechanism of its anti‐tumor activity.^234^ Mirzaaghaei et al. found that the combination of EGCG and silybin can regulate the expression of pro‐angiogenic miRNAs in endothelial cells with tumor cells.^235^ It can also coordinate with DNA methyltransferase and histone deacetylase inhibitors to upregulate E‐cadherin expression and inhibit the migration and invasion of human NSCLC cells.^236^ Am80 is a synthetic vitamin A, which has been used as a new drug in patients with relapsed and stubborn acute promyelocytic leukemia. EGCG binds to Am80 by downregulating the level of non‐histone acetylation by downregulating HDAC‐4, HDAC‐5, and HDAC‐6, and stimulating the apoptosis of human lung carcinoma cells.^237^ Berberine can inhibit N‐acetyltransferase activity in A549 cells and was found to show a negative correlation between dose and time to some extent, which may be one of the mechanisms of its anti‐tumor effect.^238^ Resveratrol epigenetically regulates the expression of zinc finger protein 36 (ZFP36) in NSCLC cells. ZFP36 is an AU‐rich essential protein that binds to the 3’‐untranslated region and promotes the decay of target mRNAs. The downregulation of ZFP36 expression leads to the stability of the target mRNAs.^239^ SirT1 is a conservative NAD^+^‐dependent deacetylase, which participates in the regulation of stress response and cell survival, and seems to play an essential role in developing cancer resistance to radiotherapy and chemotherapy, and tumorigenesis. Its expression is negatively correlated with radiosensitivity. Resveratrol regulates the apoptosis and radiosensitivity of lung cancer cells through the Sirt1/NF‐κB/Smac pathway.^240^ RHBDD1, a mammalian member of the rhomboid family of proteins, is a highly conserved intramembranous serine protease. Silencing of RHBDD1 can inhibit cell proliferation and growth in glioblastoma, colorectal, breast cancer, and hepatocellular carcinoma. Silybin inhibits the epithelial‐mesenchymal transformation of NSCLC cells by inhibiting RHBDD1 and exerts anti‐tumor effects on NSCLC cells.^241^ Reverse inducible cysteine‐rich protein with Kazal motif (RECK) is a membrane‐anchored glycoprotein that negatively regulates matrix metalloproteinase (MMP) and plays an integral role in cancer invasion and metastasis. GA inhibits experimental lung metastasis and cell invasion of A549 in a dose‐dependent manner, which is attributed to the fact that GA effectively inhibits the binding of histone deacetylase (HDAC)‐1/specific protein(Sp)‐1, and Sp1 phosphorylation is related to extracellular signal‐regulated kinase (ERK) signal transduction, resulting in the upregulation of RECK at mRNA and protein levels.^242^ In A549 cells and A549 xenografted mice, GA inhibits cell invasion and migration through a reverse‐induced cysteine‐rich protein upregulated by the kazal motif (RECK).^243^

### 3.7 Regulation of other mechanisms and the combined effect of multiple mechanisms

The HA‐CD44/RHAMM signaling pathway plays an integral role in the growth and survival of NSCLC cells. The low concentration of triptolide significantly decreased NSCLC cell growth and inhibited tumor growth in mice by targeting the HA‐CD44/RHAMM signaling axis.^244^ The Nrf2/HO‐1 signal pathway is thought to mediate cell resistance to EGCG. Metformin sensitizes NSCLC cells to EGCG by inhibiting the Nrf2/HO‐1 signaling pathway.^53^ Ginsenoside Rh2 induced A549 cell apoptosis through the Ras/Raf/ERK/p53 pathway.^245^ GA can also inhibit the viability of NSCLC cells by inducing apoptosis by inhibiting the Notch signal pathway.^246^ Tanshinone IIA increases TRAIL‐induced NSCLC cell death by selectively activating PERK/ATF4 and inhibiting the STAT3‐mediated upregulation of DR5 and downregulation of Survivin.^247^ TPL also inhibited the phosphorylation of STAT3, inhibited the transport of STAT3 into the nucleus, and reduced the expression of STAT3 target genes associated with apoptosis, migration, and cell survival, such as C‐myc, myeloid leukemia 1 (MCL‐1), BCL2, and matrix metallopeptidase‐9 (MMP‐9), thereby inhibiting cell proliferation and migration and inducing cell apoptosis.^248^

Melittin significantly inhibited the secretion of VEGF in NSCLC cells. In addition, melittin can also reduce the protein expression of VEGF and HIF‐1α. Therefore, the anti‐tumor activity of melittin may be relevant to the inhibition of the anti‐angiogenesis of VEGF and the hypoxia‐inducible factor signaling pathway.^249^ Emodin enhances cisplatin‐induced cytotoxicity by downregulating ERCC1 and inactivating ERK1/2.^250^ β‐elemene is a prospective drug to enhance tumor radiation response. Survivin and HIF‐1α are newly identified targets of β‐elemene.^251^ β‐elemene at a radiosensitizing dose could also significantly downregulate mTOR mRNA expression, HIF‐1a, and Survivin mRNA.^252^ Eukaryotic initiation factors (eIFs) play an essential role in translation initiation. Curcumin can block protein synthesis initiation by regulating eIF2a and eIF4E expressions, thus decreasing cell viability.^253^

Tumor‐associated macrophages (TAMs) play a vital part in regulating the cancer microenvironment and promoting tumor metastasis. It has two subsets: the classical subtype of activated macrophages (M1) and the alternative subtype of activated macrophages (M2). Subtype M2 macrophages stimulate a more aggressive phenotype of lung cancer cells. G‐Rh2 can convert TAM from the M2 subclass to that of M1 and prevent lung cancer cell migration.^254^ CUR can induce the cell death of ASTC‐a‐1 cells in a significant apoptotic manner through a caspase‐independent mitochondrial pathway.^25^ As a protein with a short half‐life, Mcl‐1 abundance is strictly regulated at many levels, including transcriptional, transcriptional, and post‐translational levels Tan IIA can be used as an EGFR signal inhibitor that targets the EGFR‐Akt‐Mcl1 axis, shortening the Mcl‐1 half‐life and promoting its ubiquitination, which can provide new options for NSCLC therapy.^255^

Silybin can target multiple cytokine‐induced signaling pathways, downregulate the expression of iNOS in NSCLC cells,^256^ and regulate the expression of iNOS and cyclooxygenase 2(COX2) in lung epithelial LM2 cells of tumorigenic mice by regulating the signals mediated by TNF‐α and interferon‐γ.^257^ Nitric oxide (NO) signaling plays a significant role in cancer angiogenesis and is positively correlated with the occurrence and development of lung cancer. Silybin exerts most of its chemopreventive effects and vascular prophylaxis roles by inhibiting the expression of nitric oxide synthase (iNOS) ^258^ and COX‐2 in lung tumors. These two enzymes promote the growth and progression of lung tumors by inducing VEGF expression.^66^ The overexpression of COX‐2 is usually associated with human NSCLC and participates in tumor invasion, angiogenesis, proliferation, and anti‐apoptosis. Triptolide inhibits COX‐2 expression through COX‐2 mRNA stability regulation and post‐transcriptional regulation.^259^ Resveratrol can enhance the anticancer effect of paclitaxel on NSCLC cells in vitro by reducing COX‐2 expression at mRNA and protein levels, indicating that resveratrol has the potential to be used as a promising sensitizer for PA.^260^

## 4 DILEMMAS AND POSSIBLE SOLUTIONS

Although compounds from microorganisms, marine organisms, plants, and animals are often used to treat cancer in clinical practice, they are often limited by specific inherent characteristics. For example, CPT, curcumin, GA, tanshinone, TPL, EGCG, resveratrol, artemisinin, and other compounds have poor water solubility, low biocompatibility, low oral bioavailability, instability, and poor pharmacokinetic properties, which hinder their clinical application.^21^, ^130^, ^204^, ^261^, ^262^, ^263^, ^264^, ^265^ In addition, the yield of natural separation from natural products is low.^106^

More importantly, the use of these natural compounds often produces severe side effects. For example, side effects of camptothecin include myelosuppression, nausea, vomiting, stomatitis, abdominal pain, fatigue, diarrhea, peripheral neuropathy, and hair loss.^21^ Adriamycin use may result in serious adverse events, especially myelosuppression, leading to severe neutropenia, cardiotoxicity, and septicemia.^90^ The side effects of mitomycin include myelosuppression, nausea, vomiting, diarrhea, stomatitis, rash, fever, and general discomfort, while rare but potentially severe adverse events include hemolytic uremic syndrome, hemolysis, neurological abnormalities, renal failure, and interstitial pneumonia.^21^ Excessive cytotoxicity causes some of the side effects of EbB, such as abdominal pain and diarrhea, nausea and vomiting, and sensory neuropathy.^98^, ^100^ The clinical application of TPL is usually limited by its severe toxicity.^263^ Intravenous injection of BBR solution can lead to fatal adverse cardiovascular effects.^266^

Active efforts are being made to improve therapeutic effectiveness further using drug delivery strategies that extend the exposure time of the drug to the tumor, such as through structural modification and innovative preparation methods, alternative parenteral dosage forms, and administration regimens to improve oral bioavailability.^20^, ^204^, ^263^, ^265^, ^267^ An effective combination of conventional chemotherapy agents based on nanotechnology can be used to achieve effective treatment of tumors with low toxic side effects. Research in this field has gained importance for the development of cancer treatments and their clinical application. The main advantages of nanodrugs are as follows: (a) improve the water biocompatibility and solubility of drugs; (b) surface‐modified nanoparticles prolong the tolerance time of anticancer drugs in vivo; (c) the precise accumulation of chemotherapeutic drugs with payload in the body through localization strategies; (d) stimulus‐response release; (e) reduction of toxic and side effects on normal cells and tissues.^261^

In NSCLC cells and Swiss albino mice, gelatin nanoparticles (GNP) loaded with resveratrol (RSV) exert a higher anticancer effect than free RSV. RSV‐GNPs synergistically inhibit cell cycle progression and constitutive NF‐kB activation, and induce the apoptosis of NSCLC cells. GNP has a high loading efficiency and superior efficacy in NCI‐H460 cells, making it an ideal method of transferring RSV.^268^ Gelatin (GEL) or hyaluronic acid (HA) nanoparticles aggregated through dihydroartemisinin showed higher anticancer proliferation activity than natural DHA in A549 cells, which may be because hydrophilic GEL or HA nanoparticles have greater water dispersion ability after aggregation, which can be used to increase the therapeutic effect of anticancer drugs.^269^ The magnetic nanoparticles PLGA‐PEG‐Fe3O4 loaded with silybin can also effectively inhibit the expression of the hTERT gene and the proliferation of lung cancer cells.^270^ Organic/inorganic composed of berberine (BER), hydride nanoparticles (NPs), and zinc oxide (ZnO), have been developed for the therapy of lung cancer. These NPs can improve the antiproliferative effect based on the efficacy of chemical photothermal therapy, and do not cause severe hepatotoxicity, nephrotoxicity, and blood toxicity, as shown in blood tests conducted on rats after intravenous administration.^271^ In addition, transferrin (Tf) receptors are usually overexpressed in cancer cells. Artemisinin and its analogs enhance cancer cell delivery through human serum transferrin adducts and exert distinct anticancer effects on tumors with few side effects on normal cells.^272^

Carbonic anhydrase IX (CAIX) is an enzyme expressed on the surface of lung cancer cells, but its expression in normal lung cells is limited. The anti‐CAIX antibody coupled with liposome has the characteristics of uniform particle size distribution and continuous release, which can significantly increase the uptake of cells and tumor spheres, thus enhancing the cytotoxicity of TPL in CAIX positive cancer cells. Anti‐CAIX antibody modified liposome TPL delivered through the lung not only inhibited tumor growth more effectively than other non‐targeted TPL preparations. And the survival time of mice with orthotopic lung tumors was prolonged to the greatest possible extent.^273^ Polyethylene glycol (PEG) modified long cycle BBR liposomes showed a uniform morphology, storage stability and continuous release behavior in vitro, and the liposome form led to a significant increase in circulatory retention of BBR compared with the solution. In tumor‐bearing mice, BBR liposomes selectively increased the concentration of BBR in the spleen, lung, liver and tumors, while distribution in the kidney and heart was at a lower level. Long‐term administration of BBR liposomes has been proven to be effective and safe in inhibiting tumor growth in nude mice.^266^

Nano‐polymer‐drug coupling has many significant advantages. The combination of natural products and other drugs may exert a more significant anticancer effect on lung cancer. Hyaluronic acid/actoferrin multilayer‐coated lipid nanoparticles can deliver rapamycin and berberine to lung cancer cells. In vivo studies on tumor‐bearing mice have shown that their therapeutic activity is better than free drugs.^274^ Liquid crystal nanoparticles (LCNP), as an effective carrier for co‐delivery of pemetrexed (PMX) and resveratrol (RSV), can effectively be used to treat lung cancer. PMX‐RSV‐LCNPs inhibit tumor growth by inducing apoptosis and inhibiting angiogenesis, and has little toxicity on the liver and kidney, thus allowing its broad applicability for lung cancer.^275^ In addition, local delivery of BBR and rapamycin (RAP) to tumor cells through inhalable multi‐compartment nanocomposites also has broad applicability for the treatment of lung cancer.^276^

In addition to the application of new preparations, triptolide derivatives have recently been developed to optimize bioavailability and reduce toxicity. MRx102 (MyeloRx, Vallejo, CA) significantly reduces the activation of the cell proliferation, Wnt pathway, migration, and invasion of H460 and A549 cells. Moreover, tumor metastasis and formation were found to have been inhibited in the patient‐derived xenograft NSCLC mouse model. In short, MRx102 is an effective antineoplastic drug for the treatment of lung cancer.^277^ The anti‐tumor effect of berberine (BBR) derivative 8‐cetylberberine (HBBR) was significantly higher, compared with BBR in terms of hydrophobicity and pharmacology. Low‐dose HBBR could interfere with the expression of cyclin‐D1 and E1 to induce G1 cycle arrest and induce caspase pathway to increase apoptosis, and may inhibit the PI3 K/Akt pathway in A549 cells, thus significantly inhibiting tumor growth.^278^ 8‐acetyl tyrosine (CCOP), a new derivative of *Coptis chinensis*, induced mitochondrial‐dependent apoptosis and G0/G1 cell cycle arrest in NSCLC cells. 10 mg/kg significantly delayed tumor growth in A549 xenografted nude mice, which was more efficient than that of 100 mg/kg *Coptis chinensis*.^279^

Silybin meglumine, a water‐soluble form of silymarin that can prevent EGFR mutated NSCLC cell EMT, can also prevent the regeneration of tumors that do not respond to gefitinib, thus preventing severe tumor growth.^280^ Resveratrol is quickly acidified and sulfated by glucuronide after it is removed by the human body.^264^ Intranasal administration of concentrated preparations is an effective method of exposing the lungs to sufficient quantity of resveratrol.^281^

In short, the above schemes can improve the application of these natural ingredients to a certain extent, because most of the studies have only been conducted on plant‐derived compounds. The valuable experience gained from these plant‐derived compounds may be applied to animal toxins, microorganisms, and marine organisms, especially animal toxins, which often cause severe or even irreversible damage to normal human tissues because of their aggressive properties. In addition to the defects of these chemical products themselves, another limitation to their application is their high price. For example, the price of Epothilone is relatively high, and the synthesis of the drug poses great challenges.^97^ The whole process of introducing new drugs to the market involves discovery, preclinical studies, clinical experiments, regulatory approval, and initiation.^120^ This is a time‐consuming and expensive process that can take 10 to 15 years, while natural compounds are difficult to produce commercially as they are often complex and extensive. This makes the optimization of drug candidates and commercial production very cumbersome and expensive.^86^ To ameliorate this dilemma, firstly, the demand from companies in developing countries should be stimulated, and secondly, the provision of knowledge by universities and research institutes should be increased, particularly by encouraging partnerships between universities and the pharmaceutical industry.^282^

## 5 CONCLUSION

The natural active extracts described in this article renew hope for the treatment of lung cancer. These compounds can inhibit the growth, proliferation, and invasion of lung cancer cells by regulating various signaling pathways to promote apoptosis, enhance autophagy, and block the cell cycle. They can work in combination with a variety of chemotherapeutic drugs and radiotherapy to enhance their anticancer effects, and significantly, they can also reverse drug resistance to chemotherapeutic drugs, which is exciting because the emergence of chemotherapeutic drug resistance increases the prevalence of cancer cells and brings more significant challenges for the treatment of cancer. However, these active ingredients have some disadvantages, such as low bioavailability, poor stability, and poor water solubility, which limit their clinical application, while opening a promising and novel research field to help increase the uptake and efficacy of these drugs, especially the application and development of nanotechnology. In summary, these findings indicate that natural product‐derived drugs are promising potential anticancer therapies worthy of further study to better understand the mechanisms by which they induce their anticancer effects.

## CONFLICTS OF INTEREST

The authors declare that there are no conflicts of interest.

## AUTHOR CONTRIBUTION

Tingting Wen: Writing‐Original draft,creating images. Lei Song: Conceptualization, writing‐reviewing and editing. Shucheng Hua: Supervision, Validation, funding acquisition. All authors read and approved the final manuscript.

## Data Availability

Data sharing not applicable to this article as no datasets were generated or analyzed during the current study.
